# Treatment effects of reverse total shoulder arthroplasty – a simple method to measure outcomes at 6, 12, 24 and 60 months for each patient

**DOI:** 10.1186/s12891-020-03427-7

**Published:** 2020-06-22

**Authors:** Joerg Huber, Ulrich Irlenbusch, Max J. Kääb, Falk Reuther, Georges Kohut, Andy Judge

**Affiliations:** 1 Department of Orthopedics, Stadtspital Triemli, Birmensdorferstr. 497, 8063 Zurich, CH Switzerland; 2 Orthopedic Clinics, Marienstift, Wachsenburgallee 12, D 99310 Arnstadt, Germany; 3 Sportorthopädicum Straubing, Bahnhofplatz 27, D 94315 Straubing, Germany; 4 DRK Kliniken Köpenick, Salvador-Allende-Strasse 2-8, D 12559 Berlin, Germany; 5 Clinique generale, Rue Hans Geiler 6, 1700 Fribourg, CH Switzerland; 6 Musculoskeletal Research Unit, Translational Health Sciences, Bristol Medical School, University of Bristol, Learning & Research Building, Southmead Hospital, Bristol, BS10 5NB UK; 7 Nuffield Department of Orthopedics, Rheumatology and Musculoskeletal Sciences, University of Oxford, Windmill Road, Headington, OX3 7LD UK; 8 MRC Lifecourse Epidemiology Unit, Southampton General Hospital, University of Southampton, Southampton, SO16 6YD UK

**Keywords:** Cuff arthropathy, Reverse shoulder arthroplasty, Treatment effect, Outcome, Confounders

## Abstract

**Background:**

Although shoulder arthroplasty is less common than knee or hip arthroplasty, the number of procedures being performed is increasing rapidly. The treatment effect is a simple method to measure outcome of joint replacement. The method was applied to measure results of total hip/knee arthroplasty but not yet for shoulder arthroplasty.

**Methods:**

Included were patients with unilateral cuff arthropathy (Hamada grades > = 2) treated with reversed total shoulder arthroplasty (RSA) in this prospective multicenter study. The patients were assessed with the ASES questionnaire. The treatment effects (TE) was calculated for each patient. TE = score reduction/baseline score. A positive TE means amelioration, TE = 0 unchanged, and a negative TE means worse. The primary aim was to calculate the TE’s for RSA at 6, 12, 24, and 60 months postoperatively. The secondary aim was to analyze the influence of confounders (preoperative Hamada grade, age, gender, dominance, side of the affected shoulder, general co-morbidities measured using ASA grade).

**Results:**

Two hundred three patients were included for this analysis of whom 183 patients had a complete 2 year follow up. Two years postoperatively the mean ASES score augmented significant from 20.5 to 78.7 (*p* < 0.001). The 2 year TE’s ranged from 1 to 0.09. We had no patient with a negative TE. A higher Hamada grade was associated with better TE’s (Hamada grade 4+ vs. 2, *p*-value 0.042). For age and dominant side there were weak associations where those aged 80+ and dominant side had better TE’s. The patients with higher ASA grade had lower TE’s (ASA grade 4+ vs. 1, *p*-value 0.013). The mean TE’s were 0.77 at 6-months, 0.81 at 1 year, 0.76 at 2 years and 0.73 at 5 years.

**Conclusions:**

The outcome for reverse shoulder arthroplasty can be measured with the treatment effect method; the 2 years TE’s vary from 1 to 0.09. The mean treatment effects change little in the first five postoperative years (from 0.73 to 0.81). The confounders for better TE’s were: higher severity of cuff arthropathy (Hamada grade 3, 4 and 5), less co-morbidities (ASA Grade 1), higher age (80+) and dominant side. Gender did not influence the 2-year TE’s.

**Trial registration:**

Comité intercantonal d’éthique (Jura, Fribourg, Neuchâtel), number 01/2008**,** 24.09.2008.

## Background

Although shoulder arthroplasty is less common than knee or hip arthroplasty (in 2015 there were 83,886 primary hip arthroplasties, 94,023 primary knee arthroplasties, compared to 5221 primary shoulder arthroplasties in the UK NJR) [1] the outcome seems to be just as successful or even better in reducing pain and ameliorating shoulder function [2–7] compared to other total joint arthroplasties. From 1991 to 2010 the number of shoulder arthroplasties increased very rapidly with 98% for shoulder hemiarthroplasty and 393% especially for reverse total shoulder arthroplasty (RSA) in the New York State [8]. In California, a similar trend was found with the incidence for shoulder arthroplasties rising from 6.1/100′000 insured persons to 13.4/100′000 persons in a large cohort of an integrated healthcare system [9].

Reverse total shoulder arthroplasty (RSA) is a biomechanical unique concept of replacement surgery in the shoulder successfully used in elderly patients with cuff tear arthropathy [10]. The underlying concept was to reverse the “ball and socket” principle of the shoulder joint to lengthen the lever arm for the deltoid muscle and the rotator cuff [10] and was first described 1994 by Grammont [11]. The type of prosthesis used in this study (Affinis® inverse, Fa Mathys, Bettlach, Switzerland) was developed and introduced to the market in 2007. It has been clinically and radiographically tested [12] and can be followed in the implant registries of the Netherlands, UK, AUS and NZ. The outcomes of RSA is promising and the good mid-term results are documented in different studies [6, 7, 13–16]. The long-term outcomes (> 10 years) showed a deterioration of clinical results compared to mid-term results and a prosthesis survivorship of 93% [17].

**Fig. 1:**
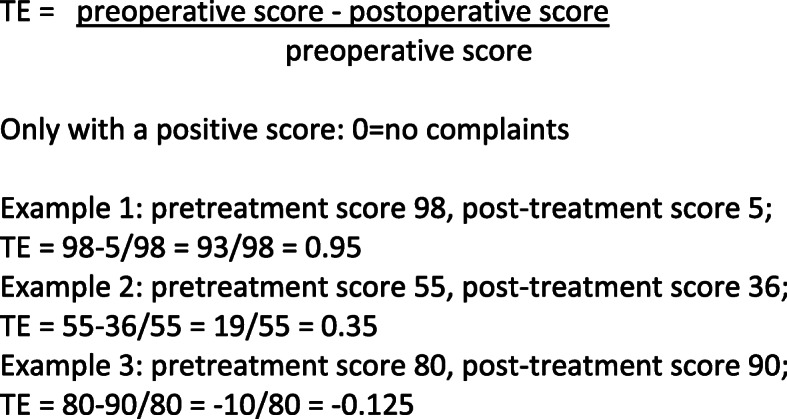
Calculating treatment effects: 3 examples

The treatment effect (TE) is a simple method to calculate the outcome for every patient individually; TE = score reduction/baseline score (see Fig. 1). The TE measures the amount of amelioration for a treatment. A positive TE corresponds to an amelioration, a TE = 0 to an unchanged situation and a negative TE to a worsening. The highest TE is 1 and corresponds to a patient without complaints after intervention [18, 19]. This kind of outcome analysis is not possible with “classical outcome” which compares the mean scores before and after treatment for a cohort. The TE method describes the variable outcome of each patient, enables closer analysis of outcome and of confounders. The method has been applied to measure outcomes of total hip/knee arthroplasty, but not yet to shoulder arthroplasty [18].

The primary aim of this study was to measure the TE’s for RSA 6, 12, 24 and 60 months postoperatively. The secondary aim was to analyze the influence of confounders (Hamada grade of cuff arthropathy, age, gender, dominant side, ASA grade) on the outcome.

## Methods

The European shoulder study group consists of five clinics specialized in shoulder surgery in three different countries (three clinics in Germany, two in France and one in Switzerland). Each clinic included their first consecutive patients in this open multicenter study. Included were patients with unilateral cuff arthropathy Hamada grade > =2 [20] who agreed to the informed consent approved by the local ethical committee. Excluded were the patients with trauma/fracture, secondary osteoarthritis, no informed consent, with rheumatoid arthritis, neoplasia, with incomplete data and who had a revision (change of basic parts of the implants) in the first 2 years.

Each patient had a primary assessment before surgery with PROM’s (patient reported outcome measurements) in paper form and a clinical/functional examination to calculate the ASES score (American shoulder and elbow surgeons score [20]) and Constant score respectively [21]. In addition, the following information were collected: sociodemographic information (gender, age), dominance, side of the affected shoulder, and American Society of Anesthesiologists (ASA) grades as score for general comorbidities. The ASA definitions were: ASA 1 normal healthy person, ASA 2 patient with mild systemic disease, ASA 3 patient with severe systemic diseases, and ASA 4 patient with severe systemic diseases that is a constant threat to life [22]. Every patient had preoperative radiological assessment with standardized x-rays (shoulder ap/scapula tangential) and MRI or CT-Scan to evaluate the Hamada grade of cuff arthropathy [20].

Each patient had reversed total shoulder arthroplasty (Affinis® inverse, Fa Mathys, Bettlach, Switzerland) in a standardized way in beach chair position with cementless fixation of the base plate of the glenoid component and non-cemented or cemented fixation of the stem. The postoperative treatment with immobilization, physical therapy and beginning of loadbearing of the arm was individual and defined by each participating clinic.

Each patient had at least one complete follow up within 2 years with identical PROM’s to calculate ASES score and a clinical examination for the Constant Score. If possible the identical PROM’s were also collected 5 years after surgery. All data were documented separately in a central register. The ASES Score was used for the outcome as described in the original publication (50% pain, 50% activities of daily living (ADL)), but for correct calculation of the treatment effect the ASES score was normalized to a score from 0 (best) to 100 (worst). The ASES has just two domains of pain and ADL, and hence was preferred to the Constant Score for the analysis, as this has too many dimensions (symptoms, ROM, force, ability to work).

### Statistical methods

The outcome is measured as treatment effects (TE = (preoperative score – postoperative score) / preoperative score). The calculations were performed for each patient at each follow up (6, 12, 24 and 60 months). The ASES Score had to be inversed (0 = best, 100 = worse) to allow us to calculate correctly the TE’s. The confounders of interest were: Hamada grade of cuff arthropathy, age, gender, dominance, side of the affected shoulder, general co-morbidities measured using ASA grades.

Descriptive statistics (mean, standard deviation for continuous variables and number, percentage for categorical) are used to exclude a selection bias by the patients that had a baseline assessment and no follow up. Box-whisker plots describe change in ASES and Constant scores over pre-operative and follow up time points. Kernel density plots describe the distribution of TE’s over different follow ups. Linear regression modeling was used to describe the association of the confounders of interest.

## Results

**Fig. 2:**
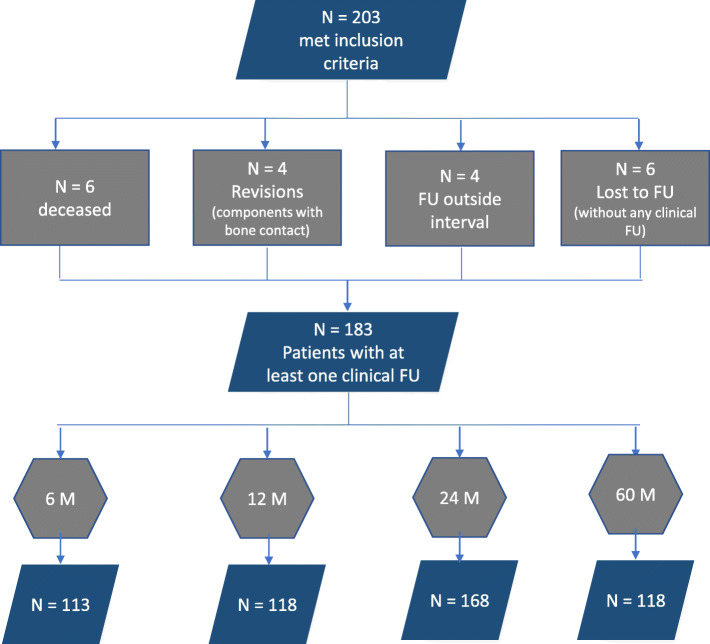
Flow chart of the patients

The study included 203 patients. Twenty had to be excluded (six for death not related to treatment, four for surgical revision of large parts with a good result in the further follow ups, and ten who were lost to follow up with a baseline assessment) (Fig. 2). This gave 183 patients with at least one clinical follow up in the first 5 years postoperatively, of whom 168 had a complete 2-year follow up ASES score (173 for the Constant Score) and 118 a complete 5-year follow up ASES score. The baseline pain, ASES and Constant scores of all included and excluded patients did not differ significantly (Table 1).

**Table 1:** Baseline characteristics of the patients at baseline and with at least one clinical follow up over 2 years

	All Patients with pre-op data	Patients with follow up data
	*N* = 203	*N* = 183
Hamada Grade		
Stage 2	55 (27.1%)	49 (26.8%)
Stage 3	43 (21.2%)	41 (22.4%)
Stage 4a; Stage 4b; Stage 5	105 (51.7%)	93 (50.8%)
Age		
Mean (SD)	74.9 (6.7)	74.7 (6.5)
Range	41.9 to 91.6	41.9 to 87.5
Gender		
Female	134 (66.0%)	122 (66.7%)
Male	69 (34.0%)	61 (33.3%)
Dominance		
Dominant	185 (91.1%)	166 (90.7%)
Non dominant	18 (8.9%)	17 (9.3%)
ASA grade		
1	15 (7.4%)	13 (7.1%)
2	22 (10.8%)	20 (10.9%)
3	69 (34.0%)	66 (36.1%)
4 and 5	97 (47.8%)	84 (45.9%)
ASES		
Mean (SD)	20.3 (12.9)	20.8 (12.8)
Range	0.0 to 63.3	0.0 to 63.3
Constant		
Mean (SD)	24.6 (13.2)	25.3 (13.2)
Range	3.0 to 67.0	3.0 to 67.0

**Fig. 3:**
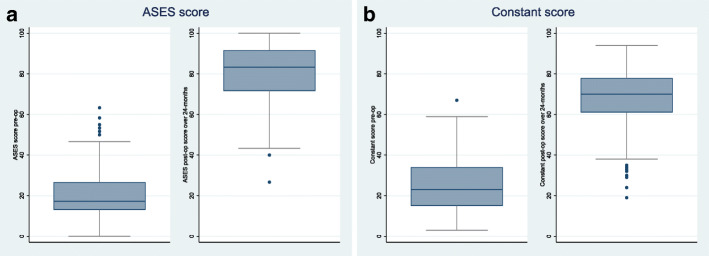
Boxplot diagrams showing change of median score for ASES score, and Constant score pre-operatively and over two-year follow up

By 2 years, the mean ASES score augmented from 20.5 to 78.7 (a difference of 58.2 95% CI (55.3 to 61.1), *p* < 0.001) (Fig. 3a), and the Constant score from 25.4 to 67.8 (a difference of 42.4 95% CI (39.9 to 44.9), *p* < 0.001) (Fig. 3b).

**Fig. 4:**
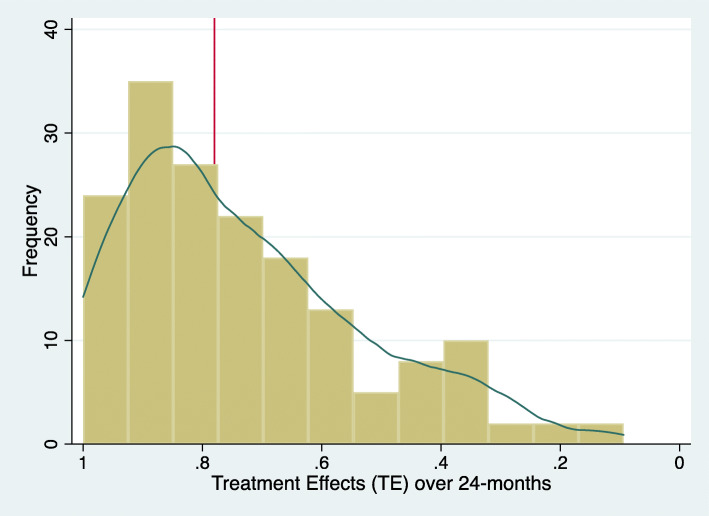
Distribution of the treatment effects (TE’s) for the two-year follow up

**Fig. 5:**
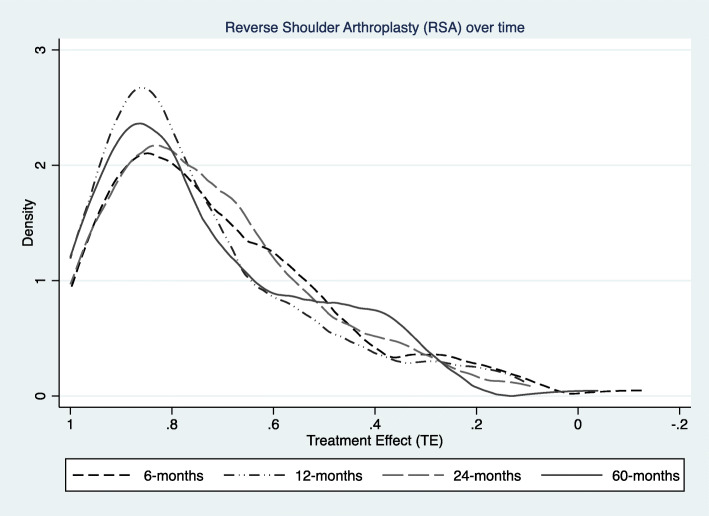
Kernel density plots of distribution of TE’s for 6, 12, 24 and 60 months follow up

The TE’s ranged from the maximum 1 to 0.09 for the 2-years follow up. We had no patient with a negative score. The median 2-years TE was 0.76 95%CI (0.73, 0.79) (see Fig. 4). Comparing different follow-up intervals we found only small differences between the distributions of the mean TE’s at 6 months, 1, 2 and 5-years (See Fig. 5), being 0.77, 0.81, 0.76 and 0.73 respectively.

There was some evidence of an association of Hamada grade on 2-year outcomes. The patients with a cuff tear arthropathy Hamada grade 4 and 5 had a mean TE that was 0.8 points higher compared to grade 2 as reference (*p* = 0.042) (Table 2).

**Table 2:** Results of linear regression model describing association of Hamada grade on TE’s

	Univariable		Multivariable	
	TE’s over 24-months		TE’s over 24-months	
	***N*** = 168		***N*** = 168	
	Coef (95% CI)	***P***-value	Coef (95% CI)	***P***-value
**Main Predictor**				
Hamada Grade				
Stage 2	REF		REF	
Stage 3	0.06 (−0.03, 0.14)	0.194	0.05 (−0.03, 0.14)	0.229
Stage 4a; Stage 4b; Stage 5	0.07 (− 0.01, 0.14)	0.07	0.08 (0.00, 0.15)	0.042
**Confounders**				
Age				
< 70	REF		REF	
70 to 80	0.01 (−0.07, 0.08)	0.894	0.02 (−0.05, 0.10)	0.546
80+	0.07 (−0.03, 0.17)	0.177	0.09 (−0.01, 0.20)	0.087
Gender				
Female	REF		REF	
Male	−0.02 (−0.08, 0.05)	0.604	−0.02 (− 0.09, 0.04)	0.518
Dominant side				
Dominant	REF		REF	
Non-dominant	−0.07 (−0.17, 0.04)	0.213	−0.10 (− 0.21, 0.01)	0.07
ASA grade				
1	REF		REF	
2	−0.03 (−0.17, 0.11)	0.673	−0.02 (− 0.16, 0.12)	0.79
3	−0.07 (− 0.19, 0.06)	0.28	− 0.09 (− 0.21, 0.03)	0.157
4 and 5	− 0.11 (− 0.24, 0.01)	0.065	−0.16 (− 0.28, − 0.03)	0.013

**Fig. 6:**
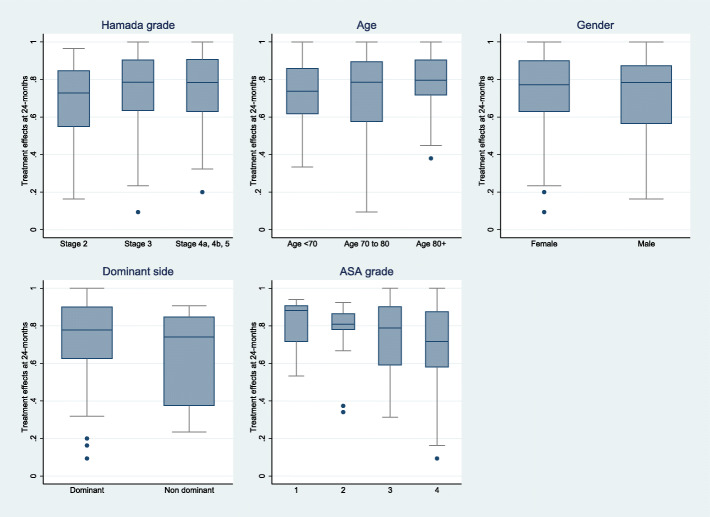
Box-plot of TE’s for main predictor (Hamada grade) and confounders (gender, age, dominance, comorbidities as ASA Score)

The TE’s differed weakly according to confounding factors of age, and dominant side but not for gender (Fig. 6). There was some difference by ASA grade whereby those with higher ASA grade had reduced TE’s (median TE in ASA 1 was 0.85 versus 0.67 in ASA grade 4). This was confirmed by the adjusted linear regression analysis (ASA grade 4+ vs. 1 difference in mean TE’s − 0.16 95% confidence interval (CI) (− 0.03 to − 0.28), *p*-value 0.013). These findings agree with the few existing literature for total hip arthroplasty where a higher ASA grade correlates with lower TE’s and age, gender have no influence [23].

## Discussion

In this study the TE’s of reverse shoulder arthroplasty were calculated for the first time using a standard score (ASES). The high treatment effects found correspond to the clinical success of RSA in patients with cuff arthropathy; mostly all patients had pain reduction and better function after surgery. The good results of earlier studies using the t-test were similar in this study (ASES Score from 20.5 to 78.7, *p* < 0.001).

The distribution of TE’s as kernel density plots demonstrate that the ameliorations can be seen already 6 months after RSA and change little in the further follow-up to 5 years (range 0.73 to 0.81 in median TE’s).

The most important factors influencing the outcome of the examined parameters were: the severity of cuff arthropathy as measured by Hamada grade, the grade of general comorbidities measured in ASA grades, age, and dominant side [22]. Interestingly there was no influence of gender.

This study is valuable because it’s a multicenter study with a defined pathology and a single treatment; most other studies have mixed indications (e.g. fracture, revision) [10, 17]. There are few multicenter studies about this type of arthroplasty with such a long follow up. Further, the data were collected in a standardized way.

The limitations of this study were: 1 the results cannot be directly compared to other shoulder studies because in this study the TE method was applied the first time to measure outcome of RSA. 2 The open design of the study with at least one follow up lead to a slightly reduced number of patients with a 2 years follow up. 3 A bias factor would be the interest and expertise of each participant surgeon in shoulder surgery and in careful patient selection to get good results. That might be a reason to explain that there was no patient with a negative TE. Existing studies also have more homogeneous populations, being single center studies, with only a single follow up time point that is not consistent between patients.

## Conclusion

The outcome for RSA in patients with cuff arthropathy can be measured with TE’s using the inverted ASES score. The excellent results also in this study may explain the fast success and rapidly increasing numbers of this treatment. The results can be seen already early 6 months after RSA with only small changes over a longer follow interval up to 5 years.

## Data Availability

Anonymized source data can be obtained from the corresponding author on reasonable request.

## Abbreviations

RSA: reversed total shoulder arthroplasty
ASES: American Society of Shoulder and Elbow Surgeons Score
TE: Treatment effect
ASA: American Society of Anesthesiologists Score
UK: United Kingdom
NJR: National joint registry (UK)
AUS: Australia
NZ: New Zealand
PROM’s: Patient reported outcome measurements
MRI: Magnetic resonance imaging
CT: Computer tomography
ADL: Activity of daily living
ROM: Range of motion
CI: Confidence interval
